# Tuning Ionic Liquid-Based Catalysts for CO_2_ Conversion into Quinazoline-2,4(1*H*,3*H*)-diones

**DOI:** 10.3390/molecules28031024

**Published:** 2023-01-19

**Authors:** Ruina Zhang, Daqing Hu, Ying Zhou, Chunliang Ge, Huayan Liu, Wenyang Fan, Lai Li, Biao Chen, Yepin Cheng, Yaoji Chen, Wei Zhang, Guokai Cui, Hanfeng Lu

**Affiliations:** 1 Innovation Team of Air Pollution Control, Institute of Catalytic Reaction Engineering, College of Chemical Engineering, Zhejiang University of Technology, Hangzhou 310014, China; 2 Zhejiang Tiandi Environmental Protection Technology Co., Ltd., Hangzhou 310012, China

**Keywords:** aminobenzonitrile, CO_2_ fixation, CO_2_ utilization, C1 chemistry, CO_2_ chemistry, CO_2_-philic, mechanism, CCUS, greenhouse gas control, carbon neutral

## Abstract

Carbon capture and storage (CCS) and carbon capture and utilization (CCU) are two kinds of strategies to reduce the CO_2_ concentration in the atmosphere, which is emitted from the burning of fossil fuels and leads to the greenhouse effect. With the unique properties of ionic liquids (ILs), such as low vapor pressures, tunable structures, high solubilities, and high thermal and chemical stabilities, they could be used as solvents and catalysts for CO_2_ capture and conversion into value-added chemicals. In this critical review, we mainly focus our attention on the tuning IL-based catalysts for CO_2_ conversion into quinazoline-2,4(1*H*,3*H*)-diones from *o*-aminobenzonitriles during this decade (2012~2022). Due to the importance of basicity and nucleophilicity of catalysts, kinds of ILs with basic anions such as [OH], carboxylates, aprotic heterocyclic anions, etc., for conversion CO_2_ and *o*-aminobenzonitriles into quinazoline-2,4(1*H*,3*H*)-diones via different catalytic mechanisms, including amino preferential activation, CO_2_ preferential activation, and simultaneous amino and CO_2_ activation, are investigated systematically. Finally, future directions and prospects for CO_2_ conversion by IL-based catalysts are outlined. This review is benefit for academic researchers to obtain an overall understanding of the synthesis of quinazoline-2,4(1*H*,3*H*)-diones from CO_2_ and *o*-aminobenzonitriles by IL-based catalysts. This work will also open a door to develop novel IL-based catalysts for the conversion of other acid gases such as SO_2_ and H_2_S.

## 1. Introduction

Large amounts of CO_2_ emissions from the combustion of fossil fuels have caused severe global climate change issues, including the greenhouse effect, global warming, and extreme climate. Carbon capture and storage (CCS) and carbon capture and utilization (CCU) are two kinds of technologies to reduce the CO_2_ concentration in the atmosphere [1]. CCS technology means that the captured CO_2_ then is pressurized and transported to a storage site, where it is injected into one of a number of types of stable geological features, trapping it for multiple hundreds or thousands of years and preventing its subsequent emission into the atmosphere [2]. Alternatively, CCU technology presents a free, abundant, and non-toxic carbon source with economic benefits to offset high capture costs, wherein CCU systems, atmospheric CO_2_ or industrially emitted CO_2_ could be recycled and converted into carbon-containing value-added chemicals and fuels [3]. Among these chemicals, quinazoline-2,4(1*H*,3*H*)-diones, regarded as pharmaceutical intermediates with a wide range of biological activity, have been successfully synthesized from CO_2_ and *o*-aminobenzonitriles. This reaction is an atom-economical route, and it has been widely investigated using kinds of catalysts, such as metal oxides, [4,5] inorganic/organic supported/grafted catalysts, [6,7,8,9] organic bases, [10,11,12,13,14,15] water, [16] etc. However, most of these reaction systems have drawbacks, such as high CO_2_ pressures, high temperatures, high catalyst loadings, long reaction times, and difficulties in recycling the catalysts. Thus, the development of alternative sustainable catalysts is of great importance for efficient CO_2_ capture and conversion.

With increasing interest in the green chemistry concept, the application of ionic liquids (ILs) is one of the most promising approaches to construct alternative catalysts for CO_2_ conversion into quinazoline-2,4(1*H*,3*H*)-diones in recent years. ILs are a kind of sustainable organic compound, which are composed of organic cations and organic or inorganic anions [17,18,19,20,21,22,23]. Typical cations include imidazolium, pyridinium, ammonium, phosphonium, and superbase-based protic cations, etc., while typical anions include carboxylates, phenolates, and aprotic heterocyclic anions, etc. ILs have been applied in CO_2_ capture and conversion as solvents and catalysts due to their unique properties, such as low vapor pressure, high chemical stability, and tunable “CO_2_-philic” structure-properties [24]. It is known that melting points of ILs are below 100 °C, and some ILs are room-temperature ILs. Thus, homogeneous catalysts could be obtained from pure ILs or IL-based mixtures. In addition, heterogeneous catalysts could also be prepared via grafting or supporting ILs on porous materials. The first IL for CO_2_ conversion has been reported in 2009 [25]. During this decade, kinds of IL-based catalysts have been developed for CO_2_ conversion into quinazoline-2,4(1*H*,3*H*)-diones with *o*-aminobenzonitriles as the raw materials, and most of them reported from China. The reported ILs with high basicity could help the transformation of CO_2_. There are several review articles that have been published for the synthesis of quinazoline-2,4(1*H*,3*H*)-diones from CO_2_ and *o*-aminobenzonitriles. Gheidari and Mehrdad et al. [26] have reviewed the recent advances in synthesis of quinazoline-2,4(1*H*,3*H*)-diones. Soleimani-Amiri et al. [27] and Zhu et al. [28] have reported the mechanisms and reaction conditions of CO_2_ with *o*-aminobenzonitrile for the synthesis of quinazoline-2,4-dione. With the increasing attention on ILs as a kind of green solvent and catalyst, it is crucial to review this field systematically from a point of design functional IL-based catalysts, according to catalyze mechanism.

**Figure 1:**
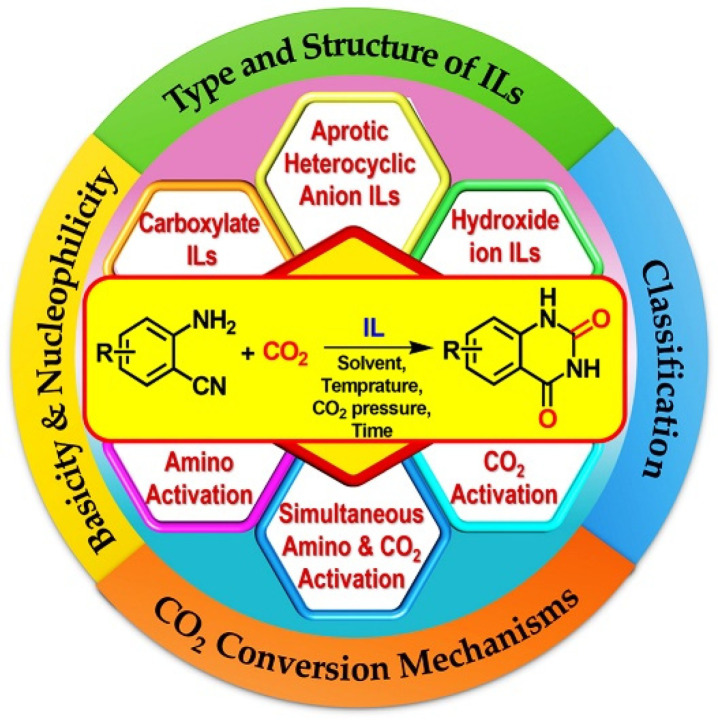
A summary on different kinds of ILs and mechanisms of CO_2_ conversion.

In this critical review, we mainly focus our attention on the tuning ionic liquid-based catalysts for CO_2_ conversion into quinazoline-2,4(1*H*,3*H*)-diones from *o*-aminobenzonitriles during this decade (2012~2022). Due to the importance of basicity and nucleophilicity of catalysts, kinds of ILs with basic anions such as [OH], carboxylates, aprotic heterocyclic anions, etc., convert CO_2_ and *o*-aminobenzonitriles into quinazoline-2,4(1*H*,3*H*)-diones via different catalytic mechanisms, including amino preferential activation, CO_2_ preferential activation, and simultaneous amino and CO_2_ activation, which are investigated systematically. Finally, future directions and prospects for CO_2_ conversion by IL-based catalysts are outlined (Figure 1).

## 2. CO_2_-Philic ILs for Efficient CO_2_ Capture

**Figure 2:**
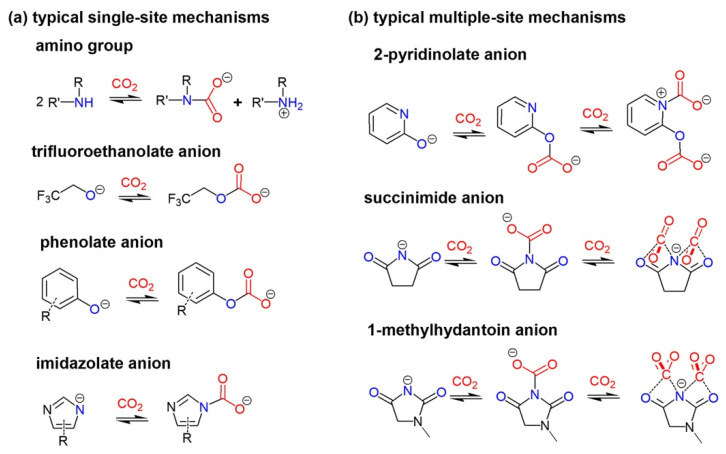
Typical structures anions and mechanisms of anion-CO_2_ reactions for (**a**) single-site reaction and (**b**) multiple-site reaction.

CO_2_-philic task-specific ILs or functional ILs used in CO_2_ capture have been developed during two decades [29,30,31,32]. The Functional ILs mainly based on functional anions include single-site anions, such as amino anions, [33,34] carboxylate anions, [35] alcoholate anion, [36] phenolate anions, [37,38] and azolate anions, [39,40,41] and multiple-site anions, such as pyridinolate anions, [42,43] imide anions, [44,45,46,47] and hydantoin anions [48,49]. The single-site anion-functional ILs result in up to a 1:1 stoichiometry absorption capacity, while multiple-site anion-functional ILs result in more than equimolar capacities. The typical anions of functional ILs and reaction mechanisms can be found in Figure 2.

## 3. CO_2_ Conversion into Quinazoline-2,4(*1H*,*3H*)-diones

The general mechanisms of the reaction of CO_2_ and *o*-aminobenzonitriles by catalysts reported in the literature can be classified into three categories: amino preferential activation, CO_2_ preferential activation, and both amino and CO_2_ simultaneous activation.

### 3.1. Amino Preferential Activation

#### 3.1.1. [OH]-Based ILs as a Catalyst

(1) [Bmim][OH]

**Figure 3:**
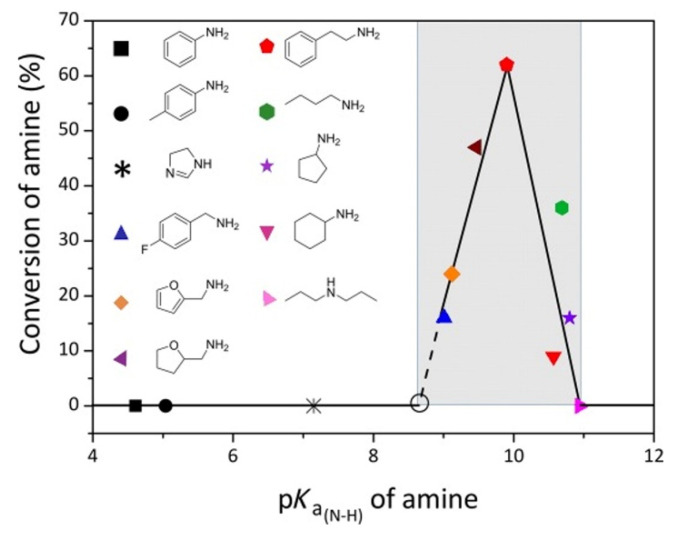
Relationship between amine conversion and pKa_(N-H)_ values. Reaction conditions: amine (0.6 mmol), DMF (2 mL), 1 bar CO_2_, 100 °C, 2 h. Reprinted with permission from Ref. [50]. Copyright 2015 Wiley-VCH.

**Figure 4:**
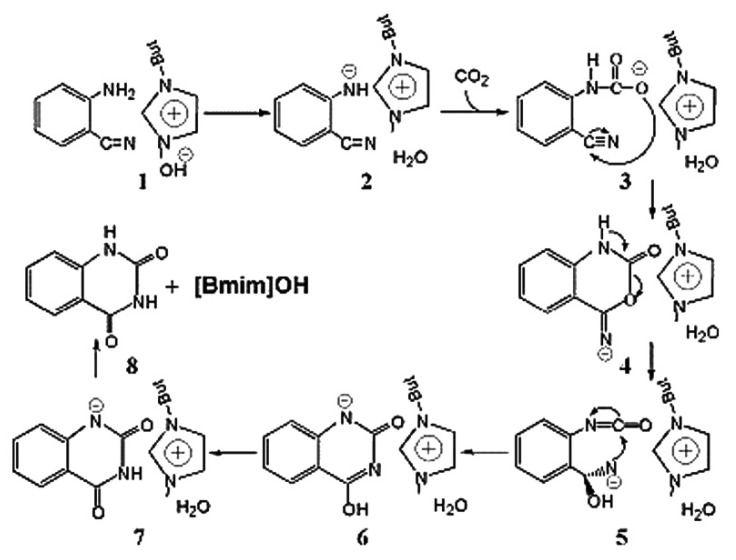
The mechanism for the chemical fixation of CO_2_ with *o*-aminobenzonitrile in the presence of [Bmim][OH]. Reprinted with permission from Ref. [51]. Copyright 2011 Elsevier Ltd.

According to the results of the study from Xu and Wang et al. [50], the amino group can react with CO_2_ when its pKa is above 8.6 (Figure 3). Therefore, as the pKa of *o*-aminobenzonitrile is relatively small, a basic catalyst is needed, which can improve the reactivity of the amino group on the *o*-aminobenzonitriles for CO_2_ conversion. The first basic IL used as a catalyst for CO_2_ conversion with *o*-aminobenzonitriles for synthesis of quinazoline-2,4(*1H*,*3H*)-diones is 1-butyl-3-methyl imidazolium hydroxide ([Bmim][OH]), which is reported by Bhanage et al. [25] They show that a remarkable activity of [Bmim][OH] can be achieved for the wide variety of substituted *o*-aminobenzonitriles. The mechanism study indicates that [Bmim][OH] only activates the amino group, and subsequently, the dehydrogenated amino group with high enough basicity to react with CO_2_. Wu et al. [51] verifies the same mechanism using a computational study. The mechanism for the chemical fixation of CO_2_ with *o*-aminobenzonitrile in the presence of [Bmim][OH] is proposed in Figure 4.

(2) Supported [OH]-based ILs

Bhanage et al. [52] report the synthesis of quinazoline-2,4(1*H*,3*H*)-diones through the carboxylative coupling of CO_2_ with *o*-aminobenzonitriles at 120 °C and 30 bar CO_2_ using the heterogeneous supported IL phase catalyst 1-hexyl-3-methyl imidazolium hydroxide supported on silica ([Hmim][OH]/SiO_2_). The influences of catalyst loading, reaction time, solvent, temperature, and pressure on the reaction system have been investigated. Their results show that lower concentrations of the [Hmim][OH] (10, 20 wt%) gives lower yields of desired product, while the optimum concentrations of the [Hmim][OH] (30 wt%) gives 85% yield. The plausible reaction pathway is similar to [Bmim][OH].

(3) Grafted [OH]-based ILs

**Figure 5:**
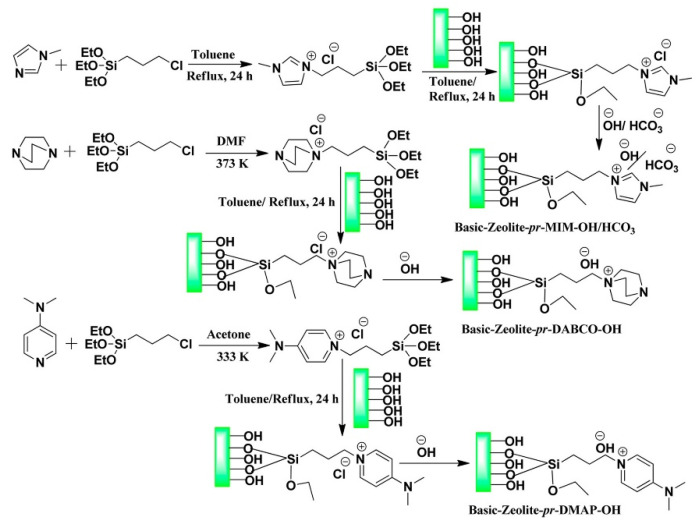
Preparation of grafted [OH]-based ILs. Reprinted with permission from Ref. [53]. Copyright 2017 American Chemical Society.

Srivastava et al. [53] report a series of [OH]-based ILs grafted on nanocrystalline Zeolites ZSM-5 for the synthesis of quinazoline-2,4(1*H*,3*H*)-diones at 150 °C and 35 bar. These grafted [OH]-based ILs catalysts have been synthesized through multiple steps, including quaternization, grafting, and [OH] anion exchange (Figure 5). They investigate the influence of solvents on the performance of grafted catalysts. The results show that the reaction has proceeded well in DMF or DMSO, while it did not take place in nonpolar solvents (toluene) and aprotic polar solvents (acetonitrile). However, only a low product yield is obtained in a polar protic solvent (methanol), while moderate product yield is obtained in H_2_O. The plausible reaction pathway is also similar to [Bmim][OH].

#### 3.1.2. Carboxylate-Based ILs as Catalysts

(1) [OAc]-based ILs

**Figure 6:**
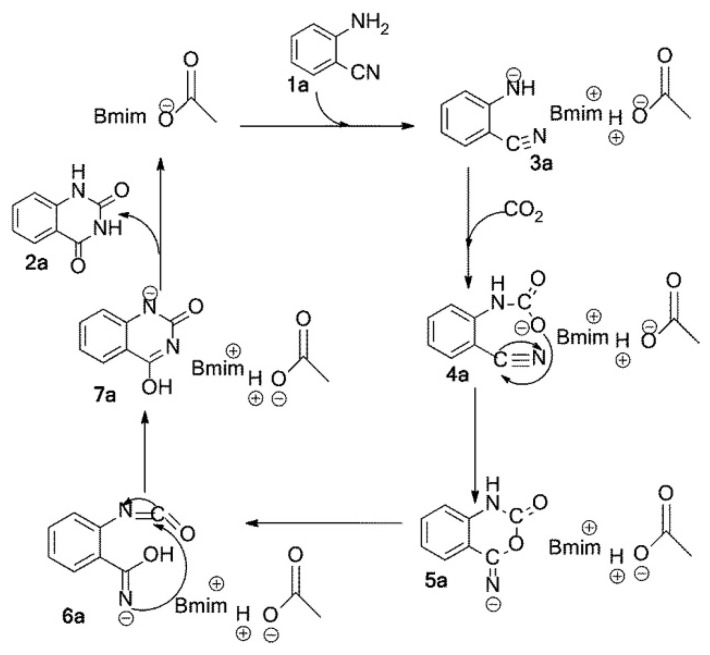
A plausible mechanism for the reaction of CO_2_ and *o*-aminobenzonitrile catalyzed by [Bmim][OAc]. Reprinted with permission from Ref. [54]. Copyright 2014 Royal Society of Chemistry.

Han et al. [54] reports that 1-butyl-3-methylimidazolium acetate ([Bmim][OAc]) can act as both solvent and catalyst for the conversion of CO_2_ and *o*-aminobenzonitriles into quinazoline-2,4(1*H*,3*H*)-diones at atmospheric pressure of CO_2_ with high yields. Using DMF as the solvent, the results show that the yield of product increases with the increased amount of the IL, indicating that the IL is an active catalyst for the reaction. A plausible mechanism is proposed as depicted in Figure 6. After the acetate anion [OAc] captures a proton from the amino group, the activated amino group reacts with CO_2_ rapidly, leading to the formation of a carbamate. Subsequently, an intramolecular nucleophilic cyclization and the rearrangement occur. The final product will be obtained from the proton transfer.

(2) Atypical carboxylate-containing ReILs

**Figure 7:**
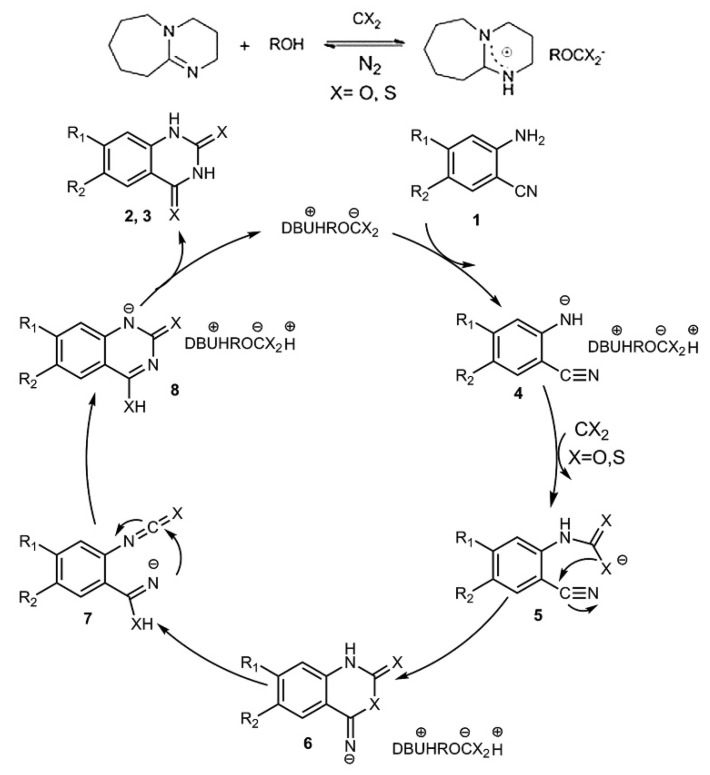
A plausible mechanism for the capture of CX_2_ (O, S) by synthesizing quinazoline derivatives in ReILs. Reprinted with permission from Ref. [56]. Copyright 2014 Royal Society of Chemistry.

The reversible ionic liquids (ReILs) have first been reported by Jessop et al. [55], where the mixture of superbase and alcohol could capture equimolar CO_2_ to form carboxylate-containing ReILs. Zheng et al. [56] have synthesized a series of ReILs as both the solvents and catalysts for the conversion of CO_2_ or CS_2_ and *o*-aminobenzonitriles into quinazoline-2,4(1*H*,3*H*)-diones or quinazoline-2,4(1*H*,3*H*)-dithiones. A plausible mechanism is proposed as depicted in Figure 7. As can be seen, the reaction mechanism using this atypical carboxylate-containing ReILs as the catalyst is similar to other mechanisms using typical carboxylate anion-containing ILs as the catalysts. The CO_2_ conversion can be performed at 40 °C and 1 bar CO_2_ with a high yield of quinazoline-2,4(1*H*,3*H*)-diones. Compared with the reaction conditions using different ILs based on anions [OH] (at 120 °C and 30 bar), [WO_4_] (at 140 °C and 1 bar), and [OAc] (at 90 °C and 1 bar), the advantages using ReILs as the catalyst can be mild conditions, high efficiency, easy separation of products, and the reusability of catalysts.

(2) [Im−CO_2_] complexes

**Figure 8:**
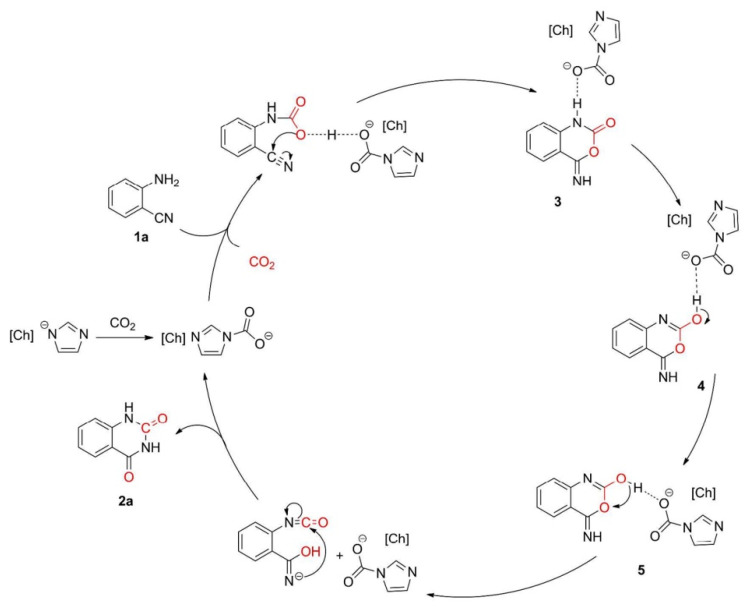
Possible reaction mechanism. Reprinted with permission from Ref. [57]. Copyright 2018 American Chemical Society.

It is known that anions play a key role in CO_2_ capture or CO_2_ utilization. However, Wang et al. [57] report that the basicity of cations affect the catalytic activity of ILs dramatically, and the hydrogen bond from cations could promote this reaction at 80 °C and 1 bar CO_2_. In their research, 7-methyl-1,5,7-triazabicyclo[4.4.0]dec-5-ene imidazolate ([HMTBD][Im]) with a higher basicity (pKa = 13.0) could improve the yield to 87%, while 1-methylimidazolium imidazolate (HMIm][Im]) with a lower basicity (pKa = 7.1) exhibits poor catalytic activity with a yield of 16%, indicating that the cation can impact this reaction dramatically. In addition, the results of the quantum-chemical calculations, NMR spectroscopic investigations, and controlled experiments indicate that in-situ generated [Ch][Im−CO_2_] is the real catalyst for the conversion of CO_2_ and *o*-aminobenzonitriles into quinazoline-2,4(1*H*,3*H*)-diones. Thereby, the possible reaction mechanism can be illustrated in Figure 8, which is similar to that of using [OAc]-based ILs as catalysts.

#### 3.1.3. Aprotic Heterocyclic Anion ILs as Catalysts

(1) Azolate-based anion IL

**Figure 9:**
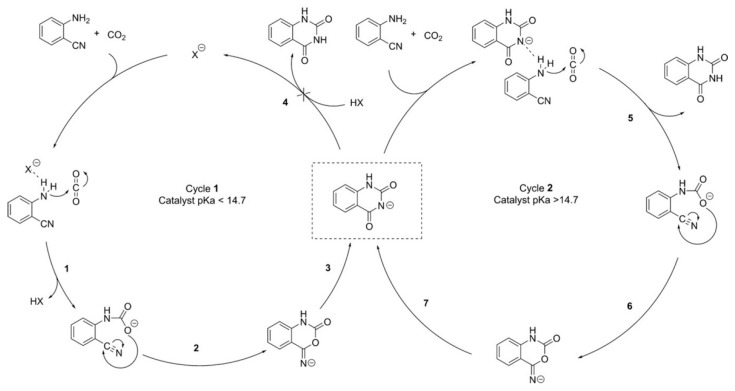
Proposed catalytic cycle for the preparation of quinazoline-2,4-diones by catalysts with a pKa < 14.7 (cycle 1) and the cycle for catalysts with a pKa > 14.7 (cycle 2). The IL cation was omitted for clarity. Reprinted with permission from Ref. [58]. Copyright 2017 Wiley-VCH.

Dyson et al. [58] investigate the relationship between the pKa of the IL anion (conjugate acid) and the yield of quinazoline-2,4(1*H*,3*H*)-dione. The results show that a linear relationship is found in the pKa range of 9.2~14.4. They have determined the acidity of the quinazoline-2,4(1*H*,3*H*)-dione product using UV/Vis spectroscopy, and the measured pKa is 14.7. Thus, all catalysts with pKa values above 14.7 only act as pre-catalysts towards the formation of the quinazolide anion IL catalysts, explaining the uniform reaction yield observed for all ILs with a pKa above this value (Figure 9). The results indicate that neutralization of the original catalyst and formation of the alternative quinazolide anion catalyst leads to the efficient CO_2_ conversion.

(2) imide-based anion IL

**Figure 10:**
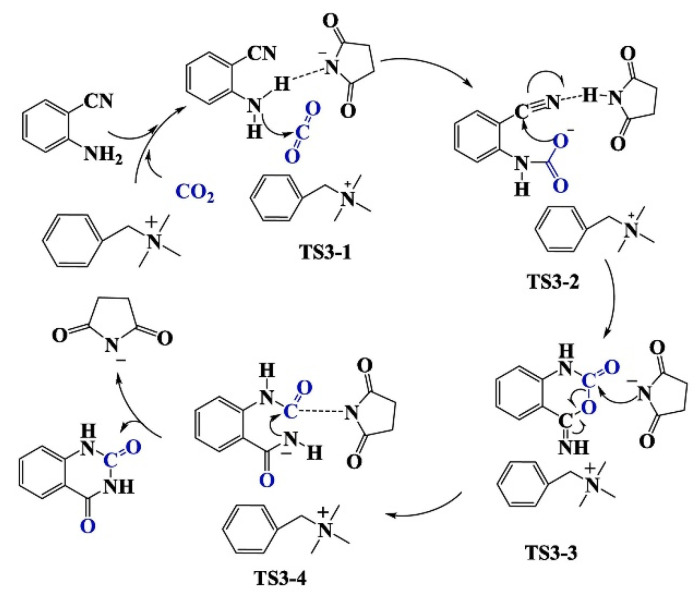
Description of catalytic mechanism. Reprinted with permission from Ref. [59]. Copyright 2022 Elsevier Ltd.

It is reported by Cui and Wang et al. [45] that tri-*n*-butylethylphosphonium succinimide ([P_4442_][Suc]) affords to introduce the CO_2_ into the IL phase even under the low concentration of CO_2_ by formation of [Suc−CO_2_] and [Suc−2CO_2_] through preorganization and cooperation. Recently, Wang and Zhang et al. [59] have reported a series of [Suc]-based ILs with different kinds of cations for CO_2_ capture and CO_2_ conversion into quinazoline-2,4(1*H*,3*H*)-diones. The results of absorption performance show that 1.45 mol CO_2_ has been absorbed by each mol of benzyltrimethylammonium succinimide ([BzTMA][Suc]) in DMSO, while only 0.69 and 0.42 mol CO_2_ per mol IL are absorbed by [Ch][Suc] and [HMIm][Suc], respectively, due to the strong interaction between active hydrogen atoms in these cations and [Suc] anions. In order to study the mechanism of CO_2_ conversion, the role of the anion/cation, the synergistic effect of the cation and anion, and the actual catalytic species have been systematically investigated based on the different catalytic species, including [BzTMA][Suc−CO_2_], [BzTMA][Suc], and [BzTMA−CO_2_][HSuc]. Since the NMR results could not totally elucidate the active species ([Suc−CO_2_] or [Suc]), the calculations have been performed. The negative charge of two O atoms in the anion of [BzTMA][Suc−CO_2_] are −0.695 e and −0.753 e, while the negative charge of the N atom in the anion of [BzTMA][Suc] is −0.793 e. As more negative charge is favorable for activating the substrate, the actual catalytic species should be [Suc] rather than [Suc−CO_2_]. Therefore, the possible reaction mechanism can be illustrated in Figure 10, including amino activation, cyclization, ring-opening, and ring-closure and catalyst regeneration.

### 3.2. CO_2_ Preferential Activation

#### 3.2.1. [Ch][OH] + Water

**Figure 11:**
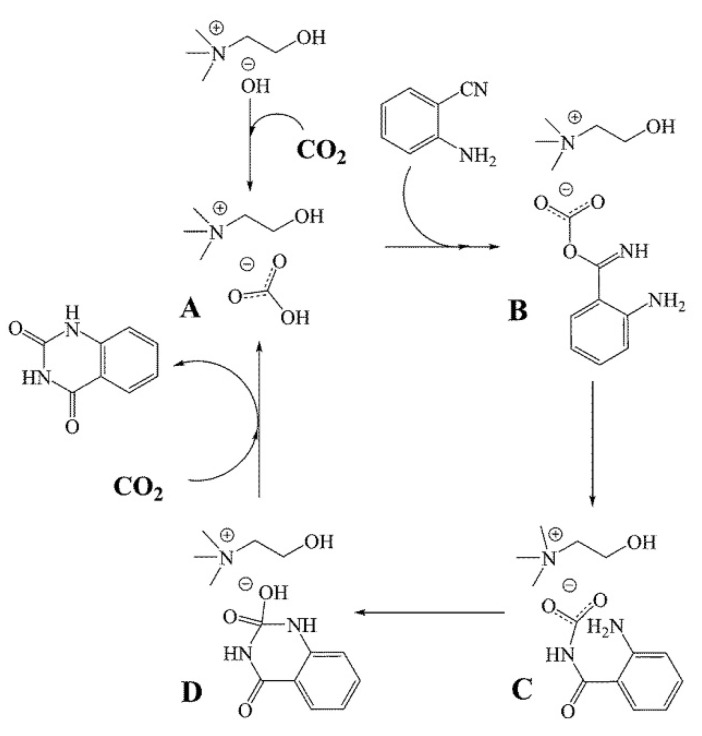
Plausible mechanism for the reaction of CO_2_ and 1a catalyzed by choline hydroxide aqueous solution. Reprinted with permission from Ref. [60]. Copyright 2014 Royal Society of Chemistry.

Ma and Han et al. [60] report that the transformation of CO_2_ and *o*-aminobenzonitriles to quinazoline-2,4(1*H*,3*H*)-diones in water can be promoted by choline hydroxide ([Ch][OH]. The results of yield vs. CO_2_ pressure show that the yield becomes independent of the pressure above 2 MPa. Additionally, the results of yield vs. temperature show that the yield can be reached 92% at 90 °C, and then remain unchanged with further-increasing temperature. The reaction mechanism is discussed and proposed as Figure 11. It is well-known that CO_2_ can form carbonic acid (H_2_CO_3_) in water. Thus, choline bicarbonate ([Ch][HCO_3_]) is formed when CO_2_ is in the aqueous [Ch][OH] solution. Subsequently, the hydroxyl H of [HCO_3_] migrates to the N atom of the nitrile group, while the O atom of [HCO_3_] nucleophilically attacks the C atom of the nitrile group. After a series of rearrangement and catalyst regeneration steps, the quinazoline-2,4(1*H*,3*H*)-dione is formed.

#### 3.2.2. NHC as a Catalyst

**Figure 12:**
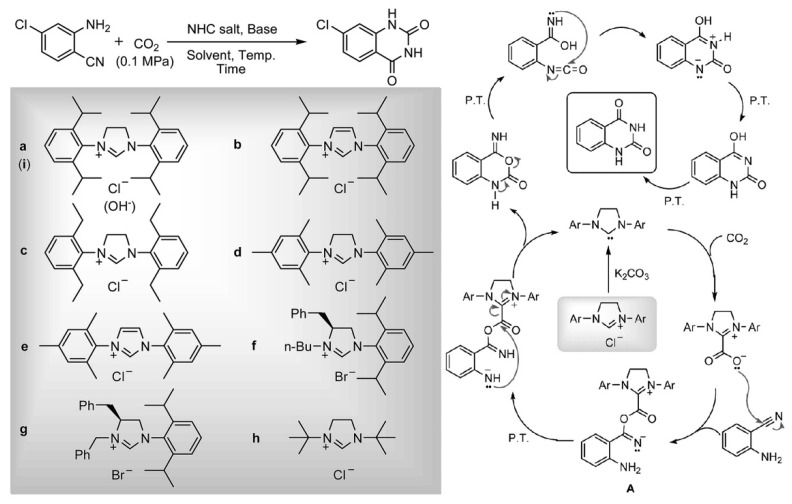
Plausible reaction mechanism for the reaction of *o*-aminobenzonitriles with CO_2_. Reprinted with permission from Ref. [61]. Copyright 2015 Royal Society of Chemistry.

Shi et al. [61] reports that the quinazoline-2,4(1*H*,3*H*)-diones can be obtained from CO_2_ and *o*-aminobenzonitriles catalyzed by N-heterocyclic carbenes (NHCs) at 120 °C and 1 bar CO_2_. The NHCs are generated from a series of imidazolium chloride in the presence of the base K_2_CO_3_ in DMSO. The plausible reaction mechanism is reported and illustrated in Figure 12. The authors suggest that NHCs are the catalytic active species, and NHCs transfer CO_2_ to quinazoline-2,4(1*H*,3*H*)-diones via the formation of NHC−CO_2_ adducts. After the nucleophilic addition of zwitterionic adducts to the nitrile group and the intramolecular cyclization, the NHCs are regenerated. Finally, quinazoline-2,4(1*H*,3*H*)diones are produced via a series of proton transfer, ring opening, and intramolecular nucleophilic addition.

#### 3.2.3. [Bu_4_P][2-MIm] as a Catalyst

**Figure 13:**
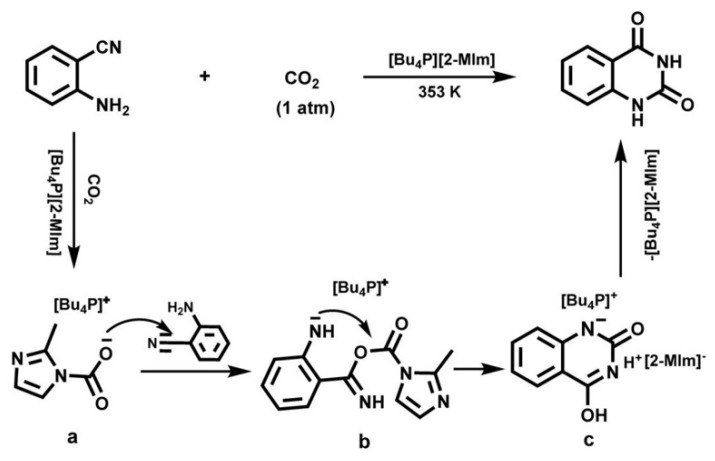
Possible pathway for the reaction of *o*-aminobenzonitrile with CO_2_, catalyzed by [Bu_4_P][2-MIm]. Reprinted with permission from Ref. [62]. Copyright 2014 Wiley-VCH.

Liu et al. [62] reports that quinazoline-2,4(1*H*,3*H*)-diones can be obtained in excellent yields from atmospheric CO_2_ and *o*-aminobenzonitriles using tetrabutylphosphonium 2-methylimidazolate ([Bu_4_P][2-MIm]) as a catalyst. A possible reaction mechanism is proposed and illustrated in Figure 13. It can be seen that the carbamate intermediate [2-MIm−CO_2_] is first generated from the reaction of CO_2_ and [2-MIm] anion. After IL nucleophilically attacks the CN group and is followed by intramolecular nucleophilic cyclization and hydrogen transfer, the corresponding quinazoline-2,4(1*H*,3*H*)-dione is obtained and the catalyst is regenerated.

### 3.3. Amino/Nitrile and CO_2_ Simultaneous Activation

#### 3.3.1. Oxylate Anion as a Catalyst

(1) [TBA]_2_[WO_4_] IL as a catalyst

**Figure 14:**
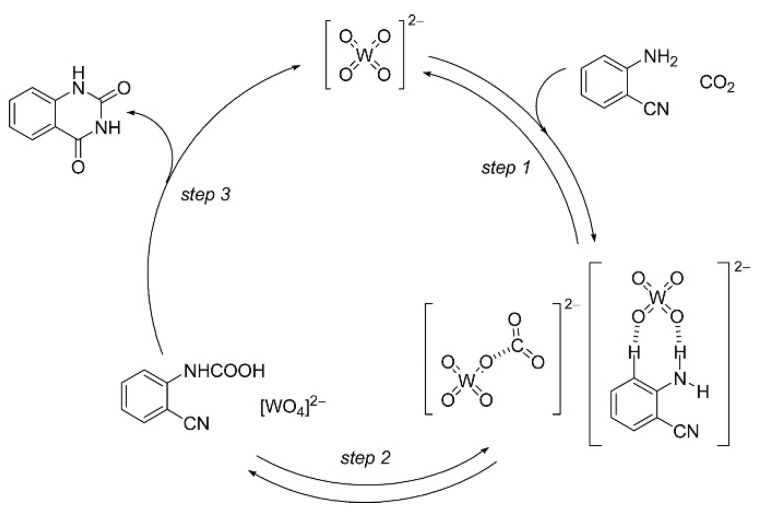
Proposed reaction mechanism for chemical fixation of CO_2_ with *o*-aminobenzonitrile catalyzed by [TBA]_2_[WO_4_]. Reprinted with permission from ref. [64]. Copyright 2014 Wiley-VCH.

Mizuno et al. [63,64] reports tetra-*n*-butylammonium tungstate ([TBA]_2_[WO_4_]) to show catalytic activities for the chemical fixation of CO_2_ with various kinds of *o*-aminobenzonitriles in N-methylpyrrolidone (NMP) to the corresponding quinazoline-2,4(1*H*,3*H*)-diones with high yields at 140 °C and atmospheric pressure of CO_2_. The proposed reaction mechanism for conversion of CO_2_ and *o*-aminobenzonitrile catalyzed by [TBA]_2_[WO_4_] is illustrated in Figure 14. In the proposed mechanism, amino and CO_2_ are simultaneously activated by [WO_4_] anion firstly, resulting in dehydrogenated amino and [WO_4_−CO_2_] active species. After the nucleophilic attack and proton transfer, the [WO_4_] regenerates. Finally, quinazoline-2,4(1*H*,3*H*)-diones are obtained through intramolecular nucleophilic cyclization and rearrangement.

(2) Alcoholate ILs as catalysts

**Figure 15:**
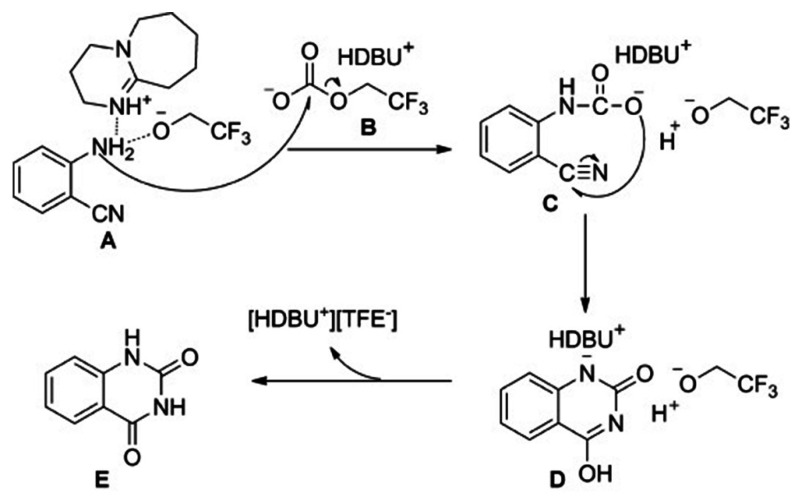
Possible reaction pathway. Reprinted with permission from Ref. [65]. Copyright 2014 Wiley-VCH.

Liu et al. [65] synthesize a bifunctional protic IL [HDBU][TFE] via the neutralization of 1,8-diazabicyclo[5.4.0]undec-7-ene (DBU) and trifluoroethanol (TFE). [HDBU][TFE] can be used as both the catalyst and solvent for the reaction of CO_2_ with various *o*-aminobenzonitriles at atmospheric pressure and room temperature, producing corresponding quinazoline-2,4(1*H*,3*H*)-diones in excellent yields. The reported possible reaction pathway can be found in Figure 15. It can be seen that [HDBU][TFE] activates both CO_2_ and the substrates simultaneously, resulting in dehydrogenated amino and [TFE−CO_2_] active species. After the nucleophilic attack, intramolecular nucleophilic cyclization, rearrangement, and hydrogen transfer, quinazoline-2,4(*1H*,*3H*)-diones are obtained and [HDBU][TFE] is regenerated. However, Mu and Liu et al. [66] investigate using systematic DFT calculations that one *o*-aminobenzonitrile molecule requires two CO_2_ molecules to yield one quinazoline-2,4-(1*H*,3*H*)-dione. One CO_2_ acts as a reactant, while another transferred to [TFE–CO_2_] acts as the catalyst. The conversion mechanism begins with nitrile activation, which is different from Figure 15. Recently, [HDBU][TFE] grafted on Fe_3_O_4_ for fixation of CO_2_ into quinazoline-2,4(1*H*,3*H*)-dione has been reported by Vishwakarm and Mahto et al. [67].

**Figure 16:**
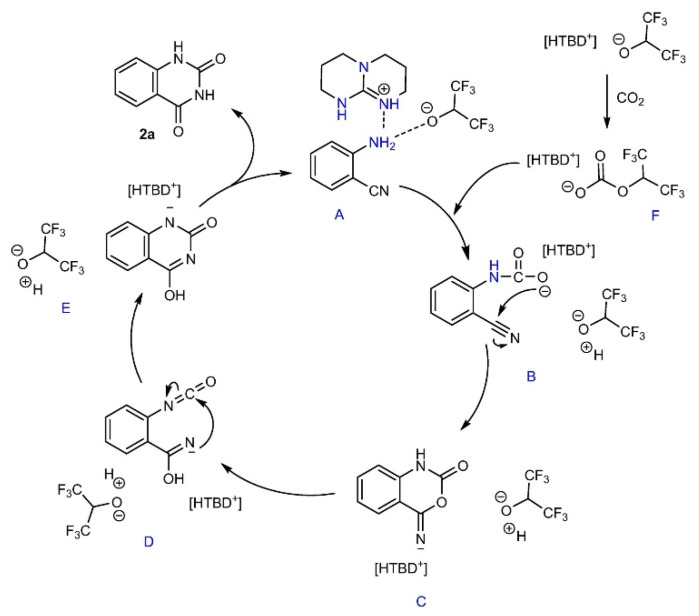
Plausible reaction mechanism for the synthesis of quinazoline-2,4(1*H*,3*H*)-diones. Reprinted with permission from Ref. [68]. Copyright 2022 Elsevier Ltd.

Bhanage et al. [68] have synthesized another bifunctional protic IL [TBDH][HFIP] via the neutralization of 1,5,7-triazabicyclo[4.4.0]dec-5-ene (TBD) and hexafluoroisopropanol (HFIP). They have found that [TBDH][HFIP] simultaneously activates *o*-aminobenzonitrile as well as CO_2_ and shows excellent performance for the conversion of *o*-aminobenzonitriles into quinazoline-2,4(1*H*,3*H*)-diones at 35 °C and 1 bar CO_2_. The proposed plausible reaction mechanism in Figure 16 for the synthesis of quinazoline-2,4(1*H*,3*H*)-diones by [TBDH][HFIP] from CO_2_ and *o*-aminobenzonitriles is similar to the mechanism using [HDBU][TFE].

(3) Phenolate ILs as catalysts

**Figure 17:**
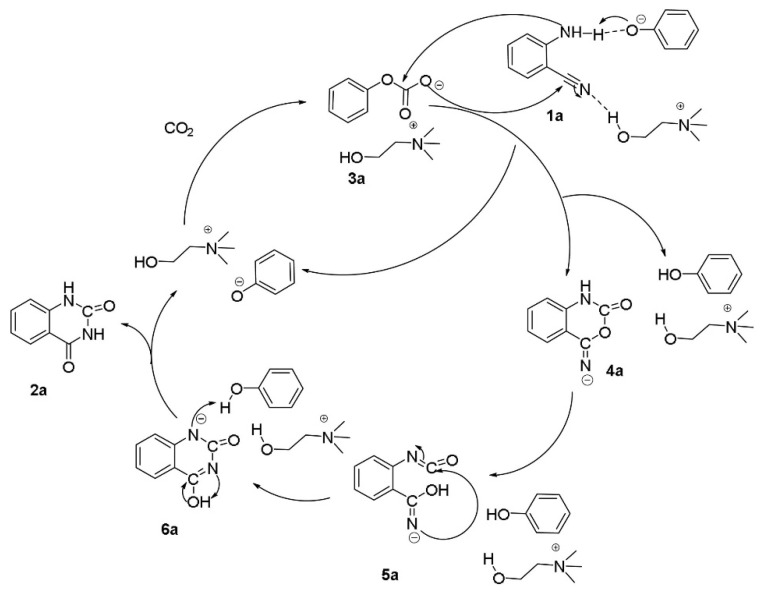
Plausible catalytic reaction mechanism. Reprinted with permission from ref. [69]. Copyright 2019 Elsevier Ltd.

Zhu and Wang et al. [69] have synthesized a series of aprotic phenolate ILs with cholinium cation and substituent phenolate anions. Among these ILs, cholinium 2,4-dichlorophenolate ([Ch][2,4-Cl-PhO]) can efficiently promote the conversion of CO_2_ to quinazoline-2,4(1*H*,3*H*)-diones at 40 °C and 1 bar CO_2_. The reported plausible catalytic reaction mechanism can be found in Figure 17. CO_2_ is activated by the phenolate anion ([PhO]), forming a carbonate anion [PhO–CO_2_]. The amino group is activated by [PhO] via hydrogen bond, and simultaneously, the nitrile group is activated by the hydroxyl group on the [Ch] cation. After intramolecular nucleophilic cyclization, rearrangement, and hydrogen transfer, quinazoline-2,4(1*H*,3*H*)-dione is obtained and [Ch][PhO] is regenerated.

**Figure 18:**
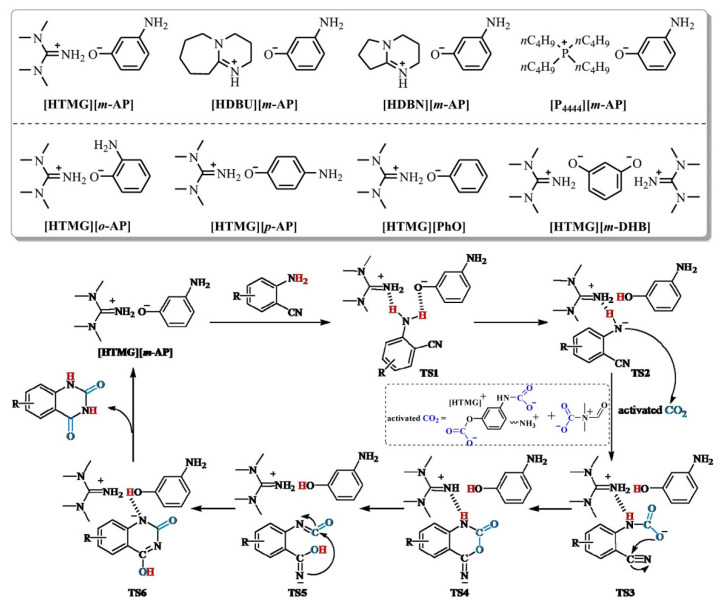
Plausible Reaction Mechanism over the [HTMG][*m*-AP]/DMF Media. Reprinted with permission from ref. [70]. Copyright 2021 American Chemical Society.

Liu et al. [70] have synthesized a series of protic ILs via the neutralization of bases and kinds of phenols. The bases include 1,8-diazabicyclo[5.4.0]undec-7-ene (DBU), 1,1,3,3-tetramethylguanidine (TMG), 1,5-diazabicyclo[4.3.0]-5-nonene (DBN), and tetrabutylphosphonium hydroxide ([P_4444_][OH]), while the phenols include *o*-aminophenol (*o*-AP), *m*-aminophenol (*m*-AP), *p*-aminophenol (*p*-AP), phenol (PhO), and *m*-dihydroxybenzene (*m*-DHB). Among these ILs, [HTMG][*m*-AP] in dimethylformamide (DMF) solvent can convert CO_2_ into quinazoline-2,4(1*H*,3*H*)-diones at 60 °C and 1 bar. A synergistic catalytic mechanism is proposed in Figure 18. As can be seen, amino and CO_2_ are simultaneously activated by [HTMG][*m*-AP] and DMF, resulting in the dehydrogenated amino, carbamate, and carbonate species. After the nucleophilic attack, intramolecular nucleophilic cyclization, rearrangement, and hydrogen transfer, quinazoline-2,4(1*H*,3*H*)-diones are obtained and [HTMG][*m*-AP] is regenerated.

(4) Carboxylate IL as a catalyst

**Figure 19:**
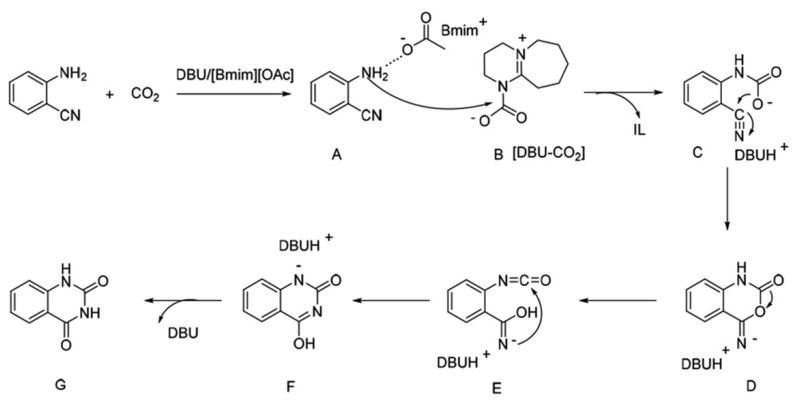
Possible reaction pathway. Reprinted with permission from Ref. [71]. Copyright 2020 Royal Society of Chemistry.

Gao and Zhou et al. [71] report that DBU/[Bmim][OAc] displays excellent performance in catalyzing the reactions of CO_2_ with *o*-aminobenzonitriles at 60 °C and 1 bar. Additionally, the mixtures containing [Bmim][OAc] and one of inorganic bases like NaOH, KOH, Na_2_CO_3_, or Cs_2_CO_3_, or organic bases like TMG, imidazole, or DBN, could catalyze this reaction, affording quinazoline-2,4(1*H*,3*H*)-diones in a yield of >78%. The reported possible reaction pathway is shown in Figure 19. Firstly, *o*-aminobenzonitrile and CO_2_ are activated simultaneously by [OAc] and DBU, respectively. After the nucleophilic attack, intramolecular nucleophilic cyclization, rearrangement, and hydrogen transfer, quinazoline-2,4(1*H*,3*H*)-diones are obtained and DBU/[Bmim][OAc] is regenerated.

**Figure 20:**
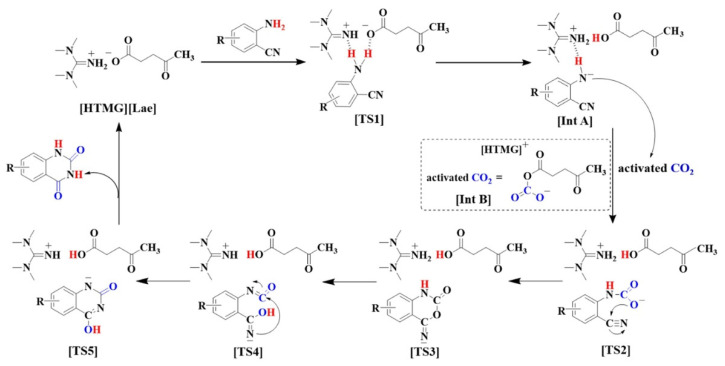
Plausible reaction mechanism. Reprinted with permission from Ref. [72]. Copyright 2022 Elsevier Ltd.

Liu et al. [72] reports that 1,1,3,3-tetramethylguanidinium laevulinate ([HTMG][Lae]) could catalyze the transformation of CO_2_ and *o*-aminobenzonitriles into quinazoline-2,4(1*H*,3*H*)-diones at 70 °C and 10 bar CO_2_ without other additives. A feasible reaction mechanism is proposed in Figure 20.

#### 3.3.2. Amino Acid Anion IL as a Catalyst

**Figure 21:**
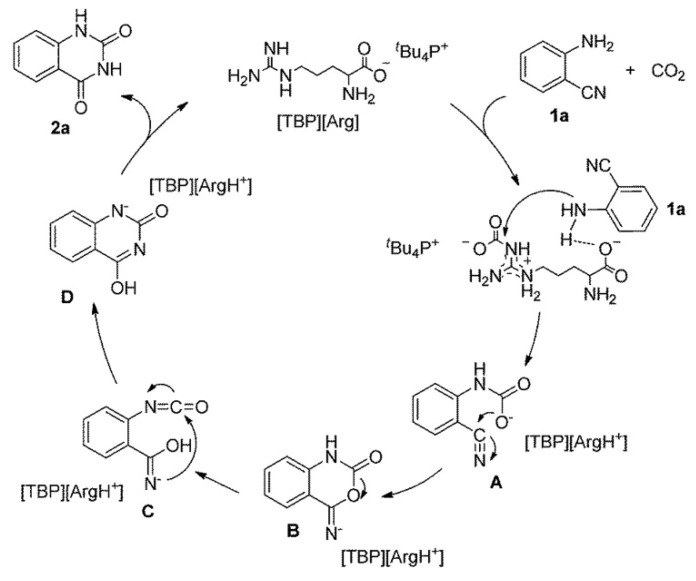
Possible pathway for the reaction of *o*-aminobenzonitrile with CO_2_ catalyzed by [TBP][Arg]. Reprinted with permission from Ref. [73]. Copyright 2015 Royal Society of Chemistry.

He et al. [73] reports that tetra-butylphosphonium argininate ([TPB][Arg]) could promote cyclization of *o*-aminobenzonitrile with CO_2_. The authors have investigated the influences of the reaction parameters, including CO_2_ pressure, reaction temperature, and time. The results showed that the yield decreases from 95% to 16% with CO_2_ pressure decreasing from 8.5 to 0.1 MPa. In addition, the comparative yields are obtained at 120 °C and 100 °C after 12 h reaction. The possible pathway for the reaction of *o*-aminobenzonitrile with CO_2_ catalyzed by [TBP][Arg] is illustrated in Figure 21. It can be seen that both the amino group and CO_2_ are initially activated by the bifunctional anion (carboxyl group and guanidine group, respectively) in [TBP][Arg]. After the nucleophilic attack, intramolecular nucleophilic cyclization, rearrangement, and hydrogen transfer, quinazoline-2,4(1*H*,3*H*)-dione can be obtained while [TPB][Arg] can be regenerated.

#### 3.3.3. Aprotic Heterocyclic Anion ILs as Catalysts

(1) Azolate anion ILs as catalysts

**Figure 22:**
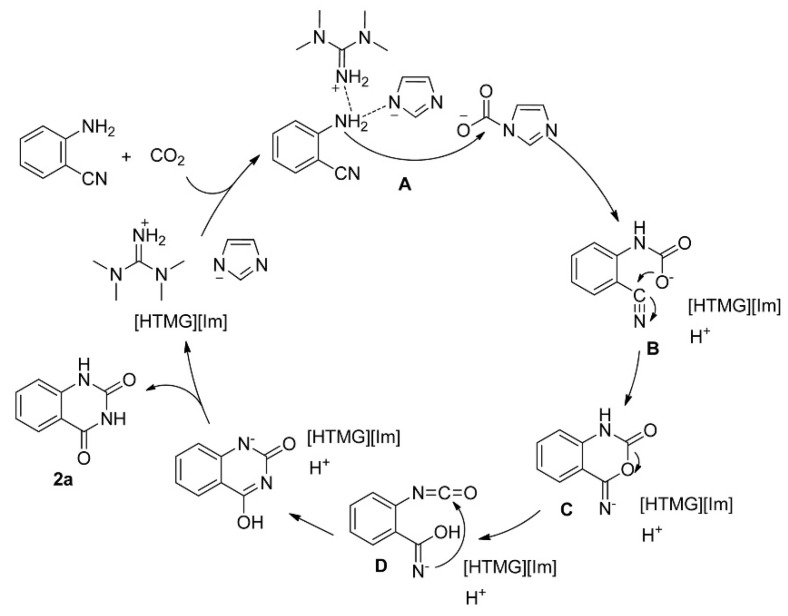
Plausible mechanism for the reaction of *o*-aminobenzonitrile with CO_2_ catalyzed by [HTMG][Im]. Reprinted with permission from Ref. [74]. Copyright 2016 Elsevier Ltd.

He et al. [74] reports a series of azolate anion ILs for the carboxylative cyclization of *o*-aminobenzonitriles with CO_2_ at 120 °C and 1 bar CO_2_. The results show that the catalytic activity follows the order of [HTMG][Im] > [HDBU][Im] > [TBA][Im] for ILs with the same anion, and the order of [HTMG][Im] > [HTMG][MIm] > [HTMG][Pyr] > [HTMG][PhO] for ILs with the same cation. In addition, the yield of 90% could be obtained at 20 °C and 1 bar CO_2_ when using 1,1,3,3-tetramethylguanidinium imidazolate ([HTMG][Im]) as the catalyst. Thus, [HTMG][Im] is chosen as a catalyst, and the plausible mechanism could be illustrated in Figure 22. With simultaneous activation of both amino and CO_2_, following the dehydrogenation, nucleophilic attack, intramolecular nucleophilic cyclization, rearrangement, and hydrogen transfer, quinazoline-2,4(1*H*,3*H*)-dione is obtained and [HTMG][Im] is regenerated.

**Figure 23:**
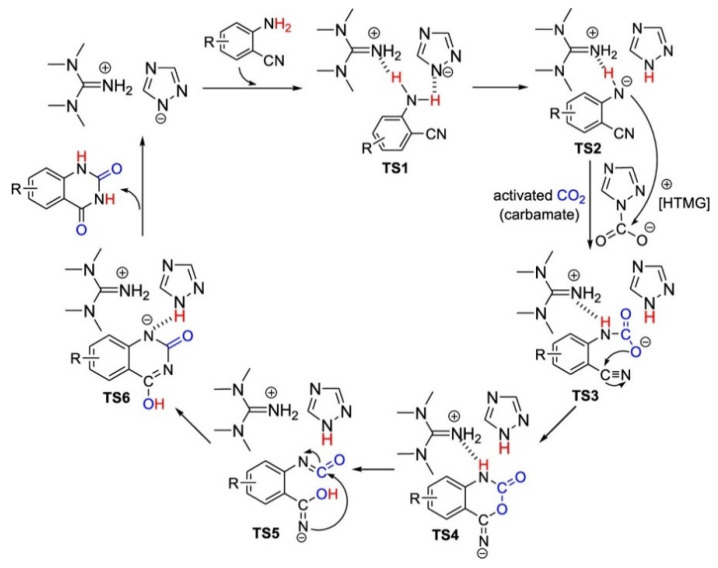
Plausible reaction mechanism. Reprinted with permission from Ref. [75]. Copyright 2020 American Chemical Society.

Liu et al. [75] have reported a series of triazolate anion ([Triz]) ILs for CO_2_ conversion into quinazoline-2,4(1*H*,3*H*)-dione, and [HTMG][Triz] has exhibited a high activity at 50 °C and 1 bar CO_2_ without any organic solvents. The plausible reaction mechanism can be found in Figure 23, which is similar to the reaction using [HTMG][Im] as the catalyst.

(2) Imide anion IL as a catalyst

**Figure 24:**
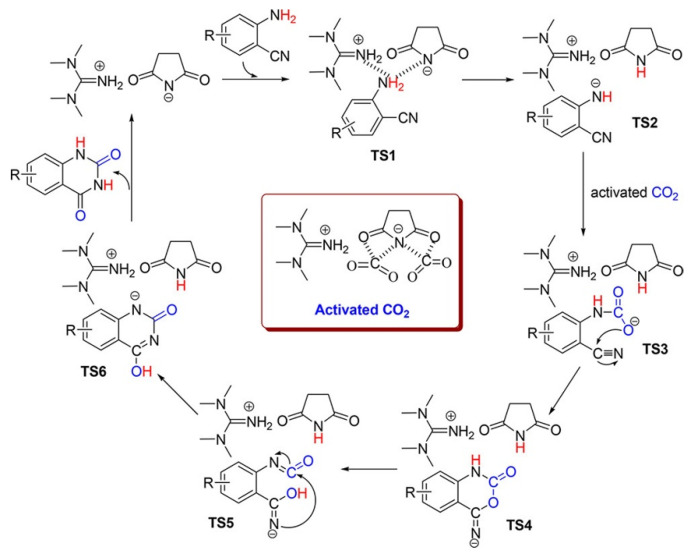
Plausible reaction mechanism. Reprinted with permission from ref. [76]. Copyright 2019 American Chemical Society.

Liu et al. [76] reports the 1,1,3,3-tetramethylguanidinium succinimide ([HTMG][Suc]) as the solvent and catalyst for the efficient transformation of CO_2_ and *o*-aminobenzonitriles into quinazoline-2,4(1*H*,3*H*)-diones at 60 °C and 20 bar CO_2_. The reported possible pathways of CO_2_ and *o*-aminobenzonitrile catalyzed by [HTMG][Suc] are illustrated in Figure 24, where both the amino group and CO_2_ are simultaneously activated.

(3) Hydantoin anion IL as a catalyst

**Figure 25:**
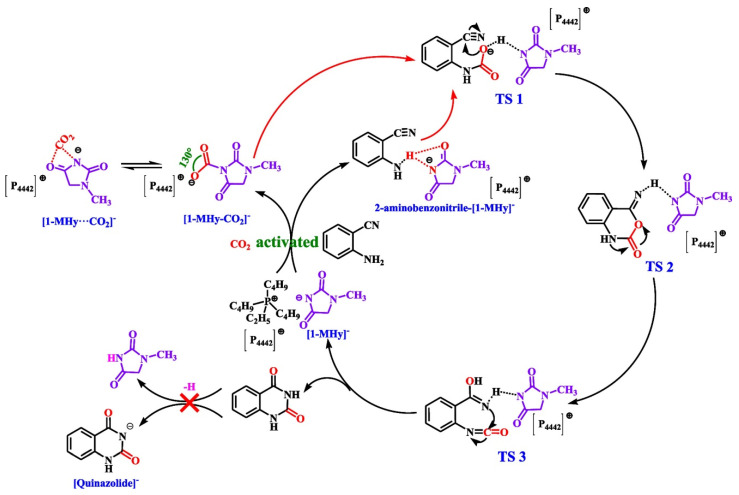
Possible reaction mechanism of CO_2_ with *o*-aminobenzonitrile catalyzed by [P_4442_][1-MHy]. Reprinted with permission from Ref. [48]. Copyright 2022 American Chemical Society.

Xu et al. [48] have synthesized a hydantoin anion-functional IL, tri-*n*-butylethylphosphonium 1-methylhydantoin ([P_4442_][1-MHy]), for efficient CO_2_ capture and catalytic conversion of CO_2_ to quinazoline-2,4(1*H*,3*H*)-dione. The results of CO_2_ capture show that the capacity of IL at 30 °C and 1 bar is up to 1.58 mol CO_2_ per mol IL, which is attributed to the multiple-site cooperative interactions. Moreover, the results of CO_2_ conversion show that the yield of quinazoline-2,4(1*H*,3*H*)-dione is 97% at 60 °C and 2 MPa CO_2_. Possible reaction mechanisms of CO_2_ with *o*-aminobenzonitrile catalyzed by [P_4442_][1-MHy] are shown in Figure 25, similar to the mechanisms of this reaction using other aprotic heterocyclic anion ILs as catalysts.

## 4. Comparison of Three Kinds of Mechanisms

### 4.1. Mechanisms Analysis

It is known that the pKa of *o*-aminobenzonitrile at 25 °C is only 0.77, [77] and insufficient nucleophilicity cannot induce nucleophilic attacks on CO_2_. Thus, it is necessary to add catalysts with basicity and nucleophilicity to improve the conversion of *o*-aminobenzonitrile and CO_2_. However, the different basicity and different nucleophilicity of catalysts result in different mechanisms. As aforementioned, there are three kinds of mechanisms of the reaction of CO_2_ and *o*-aminobenzonitriles catalyzed by ILs reported in the literature, including amino preferential activation, CO_2_ preferential activation, and both amino and CO_2_ simultaneous activation. According to the literature and our critical analysis, although the mechanisms in each group are different in detail, the steps are basically similar in general. Thus, the general reaction mechanisms for CO_2_ conversion into quinazoline-2,4(1*H*,3*H*)-diones can be summarized as follows.

For “amino preferential activation”, the anion ([X]) of IL with proper basicity first dehydrogenates the hydrogen of the amino group in the *o*-aminobenzonitrile, resulting in the active amino group with a negative charge (–HN^–^). Second, the negative-charged amino nucleophilically attacks the C atom of CO_2_, resulting in the carbamate anion (–HNCOO^–^). Third, the carbamate anion nucleophilically attacks the C atom of the nitrile group in the *o*-aminobenzonitrile, leading to an intramolecular nucleophilic cyclization. Last, the ring is rearranged along with proton transfer, resulting from the presence of tautomeric forms (–NHC( = O)– ⇌ –N = C(OH)–) or the assistance from the anion (–NH + [X] ⇌ –N^–^ + [HX]). After these four steps, product can be synthesized.

For “CO_2_ preferential activation”, the carbamate or carbonate intermediate [X−CO_2_] anion is generated first from the reaction of CO_2_ and the anion [X]. Second, the [X−CO_2_] anion nucleophilically attacks the C atom of the nitrile group in the *o*-aminobenzonitrile, resulting in a negative-charged N atom (–C = N^–^). Third, proton transfer from amino group to the negative-charged N atom results in –C = NH and a negative-charged amino group. Four, the negative-charged amino nucleophilically attacks the C atom of –COO–, leading to an intramolecular nucleophilic cyclization, accompanied by the removal of the anion [X]. Last, the ring is rearranged along with proton transfer, resulting in the target product.

For “both amino and CO_2_ simultaneous activation”, CO_2_ is first activated by the anion [X], forming a carbamate or carbonate intermediate [X−CO_2_] anion, and the amino group is simultaneously activated by [X], forming a negative-charged amino group –HN^–^ via dehydrogenation. Second, the negative-charged amino nucleophilically attacks the C atom of CO_2_ in [X−CO_2_] accompanied by the removal of anion [X], resulting in the carbamate anion (–HNCOO^–^). After intramolecular nucleophilic cyclization, ring rearrangement, and proton transfer, the target product can be obtained.

The “both amino and CO_2_ simultaneous activation” mechanism is similar to the mechanism of “amino preferential activation”. The difference between these mechanisms is the first two steps. With both amino and CO_2_ simultaneous activation, the energy consumption of the nucleophilic attack in the second step of the “both amino and CO_2_ simultaneous activation” mechanism is lower than the other one.

### 4.2. Desired Synthetic Method under Mild Reaction Conditions

Although some reported ILs as the catalysts have drawbacks (high CO_2_ pressures, high temperatures, difficulties in recycling the catalysts, etc.) as conventional catalysts (metal oxides, inorganic/organic bases, etc.), ILs still have great advantages as solvents and catalysts due to their unique tunable structures and properties. According to the aforementioned discussion, the “both amino and CO_2_ simultaneous activation” mechanism could provide an alternative opportunity to obtain the product under mild reaction conditions with low energy consumption. Through tuning the structures of ILs, CO_2_-philic task-specific ILs or functional ILs could act as the catalysts following the “both amino and CO_2_ simultaneous activation” mechanism.

These CO_2_-philic task-specific ILs or functional ILs with desired basicity could be used in CO_2_ capture. Typical functional anions and corresponding absorption mechanisms can be found in Part 2. Generally, the catalytic performance of ILs (especially anions) is affected by both cations and anions as well as the cation-anion interaction. A typical example anion is imidazolate anion ([Im]). The results of the quantum-chemical calculations, NMR spectroscopic investigations, and controlled experiments from Wang et al. [57] indicate that insitu-generated [Ch][Im−CO_2_] is the real catalyst for the conversion of CO_2_ and *o*-aminobenzonitriles following the “amino preferential activation” mechanism. Liu et al. [62] agrees that 2-methyl-substituted imidazolate ([2-MIm]) could generate [2-MIm−CO_2_] when [Bu_4_P][2-MIm] is used as the catalyst; however, the carbamate nucleophilically attacks the CN group and becomes a part of product. One possible reason is that the methyl group changes the interaction between the imidazolate anion and CO_2_. He et al. [74] show that [HTMG][Im] could simultaneously activate both amino and CO_2_, probably due to the strong interaction between the cation and anion as well as the proton on the cation. Thus, the effects of structures and interactions of anions and cations cannot be ignored. Additionally, features can be used to develop the innovative synthetic routes for the synthesis of quinazoline-2,4(1*H*,3*H*)-diones from CO_2_ and *o*-aminobenzonitriles.

## 5. Conclusions and Outlook

Because of their unique properties, ILs could be used as solvents and catalysts for CO_2_ capture and conversion into value-added chemicals. After the first IL has been reported in 2009 for the conversion of CO_2_ and *o*-aminobenzonitriles into quinazoline-2,4(1*H*,3*H*)-diones, kinds of ILs with basic anions such as [OH], carboxylates, aprotic heterocyclic anions, etc., have been developed for converting CO_2_ and *o*-aminobenzonitriles into quinazoline-2,4(1*H*,3*H*)-diones. The different catalytic mechanisms, including amino preferential activation, CO_2_ preferential activation, and simultaneous amino and CO_2_ activation, have been investigated systematically. This review is benefited for understanding the synthesis of quinazoline-2,4(1*H*,3*H*)-diones from CO_2_ and *o*-aminobenzonitriles using IL-based catalysts. However, it is clear that this field is still in its infancy. Several issues should be paid more attention to and need to be investigated further as follows.

(1) Developing IL-based heterogeneous catalysts. There are only a few examples of IL-based heterogeneous catalysts used in the synthesis of quinazoline-2,4(1*H*,3*H*)-diones from CO_2_ and *o*-aminobenzonitriles. Because of the advantages in the separation of catalysts and products from reaction system, heterogeneous catalysts based on ILs, especially functional ILs, should be developed.

(2) Conversion CO_2_ under flue gas conditions. “In situ” strategies for CO_2_ capture and subsequent conversion into value-added chemicals have been developed as potential methods for directly transforming waste CO_2_ to value-added CO_2_-based chemicals without purification. There are only a few examples of the catalysts that could be used under flue gas conditions [57]. However, more IL-based catalysts with high efficiency should be developed for CO_2_ conversion at low temperature (40~60 °C) and low CO_2_ partial pressure (0.1~0.15 bar CO_2_).

(3) Conversion mechanisms should be investigated deeply. It can be seen from the above discussion in Part 3 that reported mechanisms of ILs with anions such as carboxylate anions, aprotic heterocyclic anions, etc., in different literatures followed different routes. The main concern is whether the mechanism begins with a preferential activation or a simultaneous activation.

Generally, functional ILs with tunable structures and properties contribute an opportunity to achieve efficient CO_2_ capture and conversion into the value-added chemicals under flue gas conditions. This review article opens a door to develop novel IL-based systems for CCUS and other gases utilization.

## Data Availability

Not applicable.
